# Genome-Wide Identification of the *Xyloglucan endotransglucosylase/Hydrolase* (*XTH*) and *Polygalacturonase* (*PG*) Genes and Characterization of Their Role in Fruit Softening of Sweet Cherry

**DOI:** 10.3390/ijms222212331

**Published:** 2021-11-15

**Authors:** Zefeng Zhai, Chen Feng, Yanyan Wang, Yueting Sun, Xiang Peng, Yuqin Xiao, Xiang Zhang, Xin Zhou, Jiale Jiao, Weili Wang, Bingyang Du, Chao Wang, Yang Liu, Tianhong Li

**Affiliations:** State Key Laboratories of Agrobiotechnology, Department of Pomology, College of Horticulture, China Agricultural University, Beijing 100193, China; zhaizefeng@126.com (Z.Z.); fengc@cau.edu.cn (C.F.); yabofei1212@163.com (Y.W.); yuetingsun@126.com (Y.S.); rod@cau.edu.cn (X.P.); S20193172433@cau.edu.cn (Y.X.); 18306391375@163.com (X.Z.); zx51522zzwlwlbb@126.com (X.Z.); qingxuedanchen@163.com (J.J.); WWL0824@foxmail.com (W.W.); tzdubingyang@163.com (B.D.); wc240539@163.com (C.W.)

**Keywords:** *Prunus avium*, xyloglucan endotransglycosylase/hydrolase (XTH), polygalacturonase (PG), fruit softening, cell wall

## Abstract

Fruit firmness is an important economical trait in sweet cherry (*Prunus avium* L.) where the change of this trait is related to cell wall degradation. Xyloglucan endotransglycosylase/hydrolase (XTH) and polygalacturonases (PGs) are critical cell-wall-modifying enzymes that occupy a crucial position in fruit ripening and softening. Herein, we identified 18 *XTHs* and 45 *PGs* designated *PavXTH1-18* and *PavPG1-45* based on their locations in the genome of sweet cherry. We provided a systematical overview of *PavXTHs* and *PavPGs*, including phylogenetic relationships, conserved motifs, and expression profiling of these genes. The results showed that *PavXTH14*, *PavXTH15* and *PavPG38* were most likely to participated in fruit softening owing to the substantial increment in expression during fruit development and ripening. Furthermore, the phytohormone ABA, MeJA, and ethephon significantly elevated the expression of *PavPG38* and *PavXTH15*, and thus promoted fruit softening. Importantly, transient expression *PavXTH14*, *PavXTH15* and *PavPG38* in cherry fruits significantly reduced the fruit firmness, and the content of various cell wall components including hemicellulose and pectin significantly changed correspondingly in the transgenic fruit. Taken together, these results present an extensive analysis of *XTHs* and *PGs* in sweet cherry and provide potential targets for breeding softening-resistant sweet cherry cultivars via manipulating cell wall-associated genes.

## 1. Introduction

Softening, which is one of the irreversible ripening results, is a major determinant of shelf life and commercial quality after harvest [1]. Sweet cherries are prominent examples of high-value fruits in which softening can be commercially devastating. During the ripening process, textural changes occur which result from modifications and disassembly of cell wall structure and composition. The primary cell wall is mainly composed of cellulose, hemicellulose, pectin, structural proteins and some phenolics [2]. The solubilization of pectin and depolymerization of xyloglucan is thought to be the main modifications in cell wall responsible for the softening process, which are mediated by a set of hydrolytic enzymes such as xyloglucan endotransglycosylase/hydrolase (XTH) and polygalacturonase (PG) [3,4]. Thus, suppressing the expression of genes involved in cell wall metabolisms by genetic manipulation could be an effective way of delaying fruit softening and maintaining moderate firmness. However, currently little is known regarding the role of cell wall-modification genes in sweet cherry, which severely hampers any attempt to breed softening-resistant sweet cherry cultivars.

Xyloglucan, one of the major components of hemicellulose, has been revealed to play a critical role in the contributions of physical properties of the cell wall during plant growth [5]. Xyloglucan endotransglycosylase/hydrolase (XTH) exhibits the activity of xyloglucan endotransglycosylase that could transform new xyloglucan chains into an existing wall-bound xyloglucan or restructure existing wall-bound xyloglucan into another. On the other hand, XTH also functions as a hydrolase and exhibits xyloglucan hydrolase activity, resulting in the hydrolysis of one xyloglucan molecule. Thus, XTH is thought to be an essential enzyme involved in hemicellulose metabolism [6]. Through genomic analysis, a rising number of *XTH* genes have been identified in different plant species. In *Arabidopsis,* 33 *XTHs* with diverse organ-specific expression patterns were identified, of which five *XTHs* were mainly expressed in green siliques and two *XTHs* were expressed preferentially in stems [7]. Recently, it was showed that 26 *XTHs* in strawberry were identified, and most of them were highly expressed in roots [8]. In addition, a lot of potential *XTH* genes were also determined in *Nicotiana tabacum* [9], *Glycine max* [10], *Oryza sativa* [11], and *Hordeum vulgare* [12]. Many studies have explored the function of XTH and discovered its role in regulating fruit softening via modifying fruit cell wall. For instance, overexpression of *SlXTH1* decreased the firmness of tomato fruit, possibly through depolymerization of total sugar and xyloglucan [13]. In strawberry, studies revealed that *FvXTH9* and *FvXTH6* are required for modifying the structure of xyloglucan in the fruit cell wall, and transient expression of *FvXTH9* and *FvXTH6* accelerated the softening process of strawberry [14]. Another study showed that heterologous overexpression of Persimmon *DkXTH8* in tomato significantly promoted fruit softening, and yet exhibited more irregular and twisted cells due to the cell wall restructuring [15]. However, the identification and functional characterization of *XTH* genes in sweet cherry have not been well clarified yet.

Polygalacturonase (PG) belongs to one of the largest hydrolase families with various spatial-temporal expression profiles during plant development [16]. It has been proved that PG plays a crucial role in pectin degradation and participates in multiple developmental processes such as flower development, fruit softening and leaf abscission [17]. So far, identifications of this gene family have been reported in a wide range of plant species. Of the 45 *PG* genes determined in peach, only 16 *PGs* transcripts were detected in ripening fruit, and their expression levels in two peach varieties with different firmness are highly correlated with the softening properties [18]. In pear, a total of 61 *PGs* were identified and 28 of them showed an increment in expression during fruit storage [4]. In addition, 54 *PGs* were identified from tomato and divided into five clusters, of which cluster A and B were involved in the development of fruit and abscission zone, while cluster C, D, and F were mainly associated with the flowering development [19]. Similar analysis has been reported in other plant species, including *Arabidopsis* [20], *Brassica rapa* [21], kiwifruit [22], mango [23], and grapevine [24]. Functional characterization of the *PG* gene by genetic and molecular approaches revealed their crucial role in cell wall degradation. For examples, overexpression of *PG1* in apple trees resulted in silvery colored leaves and premature leaf shedding due to the reduced cell adhesion in leaf abscission zones [25], while downregulation of *PG1* altered firmness, tensile strength, and water loss in apple fruits [26]. In strawberry, suppressing of *FaPG1* expression led to reduction of pectin solubilization and thus increment of fruit firmness [27]. Moreover, the *Arabidopsis* polygalacturonase QRT3 was shown to degrade the cell wall of pollen mother cell during microspore development [28]. Nevertheless, studies of *PG* genes analysis and exploration of its role in fruit softening of sweet cherry have not been reported yet.

Fruit softening is also notably affected by phytohormones. Abscisic acid (ABA) has been revealed as an essential regulator of non-climacteric fruit ripening and softening. In bilberry, exogenous ABA significantly increased the expression of *VmPL*, *VmRGLyase, Vmβ-GAL1/2, VmXTH*, *VmCEL*, *VmEXP1/2/3* and accelerated fruit softening [29]. Change in the expression of ABA degradation related gene *FveCYP707A4a* influenced the expression of *FveCEL2, FvePL,* and *FvePG,* indicating that ABA is involved in the fruit softening of strawberry [30]. Ethylene, as the critical facilitator of climacteric fruit ripening, has also been reported in promote fruit softening in non-climacteric fruit sweet cherry [31], conversely, pre-harvest methyl jasmonate (MeJA) treatments could significantly inhibited the fruit softening of sweet cherry [32], but the specific molecular mechanism is not clear.

Due to the rapid softening of sweet cherry fruit, the shelf life and fruit quality are seriously influenced, and thus the cherry industry is severely restricted [33]. A possible way to tailor fruit firmness might be achieved through modulating cell wall-associated genes such as *PGs* and *XTHs*. To this end, we tapped into the genomic resources to identify potential *PGs* and *XTHs* which might be responsible for fruit softening in sweet cherry. Through analysis of the phylogenetic relationship, gene structures, and expression pattern during fruit development, we finally focused on three of these genes: *PavXTH14*, *PavXTH15* and *PavPG38*. Furthermore, transient expression assay confirmed their role in changing fruit softening via degradation of the fruit cell wall. Our results provide an extensive landscape of *XTHs* and *PGs* gene families in sweet cherry, and initial functional analysis supports the notion that *XTH* and *PG* promote fruit softening in sweet cherry.

## 2. Results

### 2.1. Identification of XTH and PG Family Genes in Prunus avium

To determine the *XTH* and *PG* family members in sweet cherry, the Arabidopsis XTH and PG protein sequences were used as queries to search against the sweet cherry genome (http://cherry.kazusa.or.jp/ accessed on 5 December 2018) by BLAST. After discarding the redundant proteins which had no characteristic amino acid residues, a total of 18 *XTHs* and 45 *PGs* were identified and hereafter named *PavXTH1-18* and *PavPG1-45* based on their locations in the reference genome (Figure S1). Detailed information of the *XTH* and *PG* genes is presented in Table 1 and Table S1, including the gene locus, gene name, protein length, molecular weight (MW), isoelectric point (PI), and signal peptide (SP). The protein length ranged from 150–639 aa for XTHs and 162–1595 aa for PGs, respectively. Additionally, the MW calculated by the ExPASy ranged from 17.43–73.43 kDa for XTHs and 17.15–175.36 kDa for PGs, while the PIs varied from 5.2–9.68 for XTHs and 5.38–9.83 for PGs. Moreover, the majority of the XTH and PG proteins contained signal peptide sequences predicted by SignalP. These variations in structural properties among *PavXTHs* and *PavPGs* suggest their multiple functions in sweet cherry.

### 2.2. Phylogenetic Analysis and Multiple Sequence Alignments of the PavXTH and PavPG Gene Family Members

**Figure 1:**
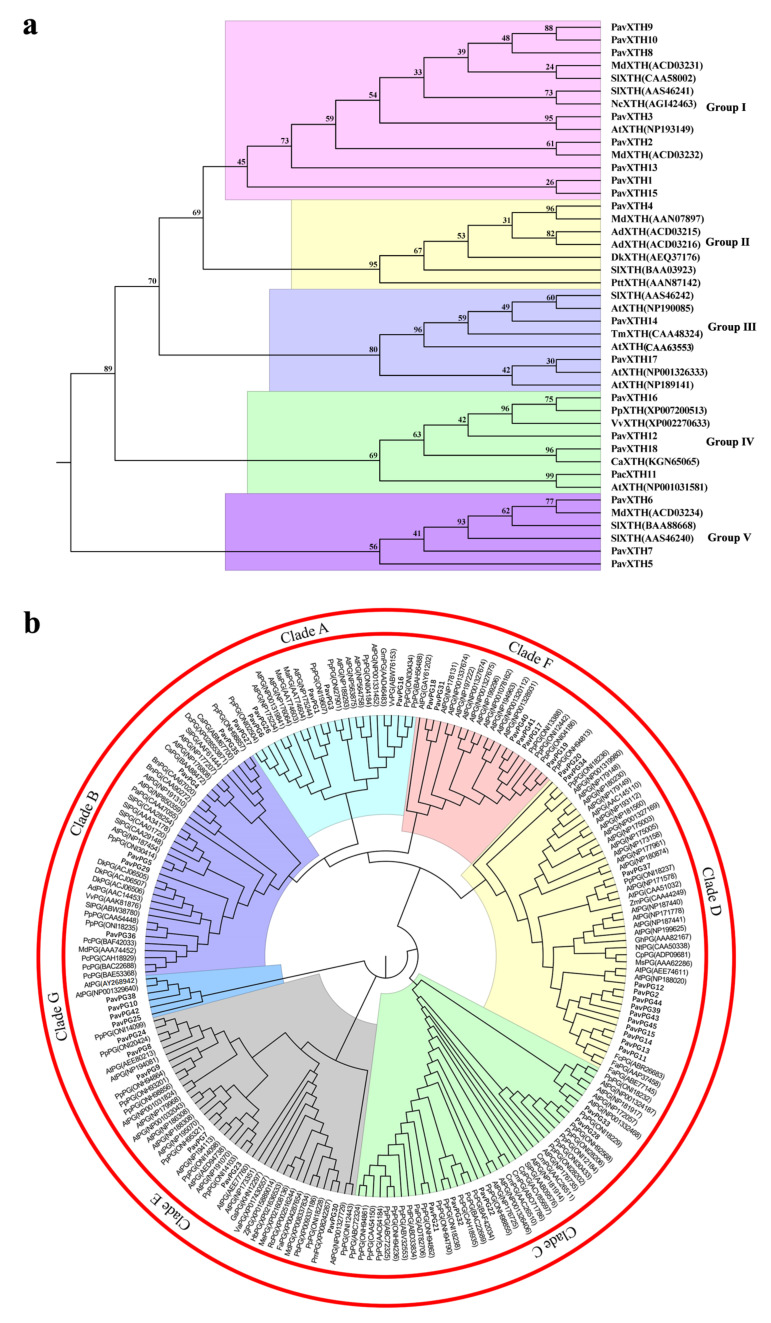
Phylogenetic relationship of XTHs (**a**) and PGs (**b**) from sweet cherry and other plants. The phylogenetic tree was constructed using MEGA 6.0 by the Neighbor-Joining method. The protein sequences of XTHs and PGs gene family members from other plants were retrieved from the GenBank database and the gene accessions are presented in the brackets.

**Figure 2:**
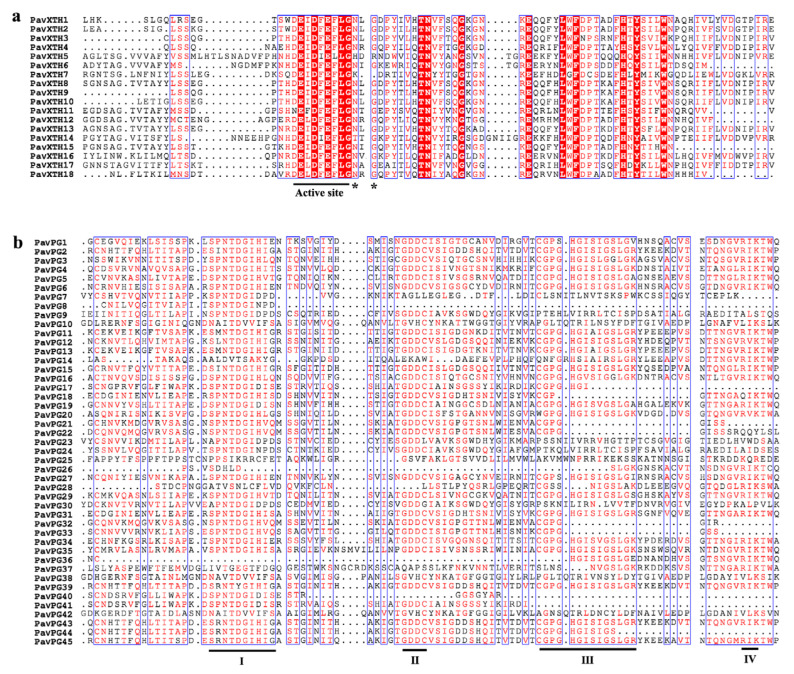
Multiple sequence alignments of the conserved domains of the PavXTHs (**a**) and PavPGs (**b**). The sequences were aligned using the Clustal X software and Espript online program. The black lines indicate the conserved domain. N-glycosylation residues are indicated as asterisks.

To further elucidate the evolutionary relationship and functional divergence of the *PavXTHs* and *PavPGs*, the rooted phylogenetic tree of 18 *PavXTHs* and 45 *PavPGs* of sweet cherry and other plants were constructed using MEGA 6.0 [34] software with a maximum likelihood (ML) method. The phylogenetic analysis showed that *PavXTH* and *PavPG* genes can be divided into five clades (Figure 1a). In the phylogenetic tree of *PavXTHs*, we observed that group I contained the largest number of genes, followed by clade IV and V. Likewise, the *PavPG* genes are divided into seven clades, among which clade D had the largest number of genes (Figure 1b). We noted that four *PavPGs* in clade G are phylogenetically close to the Arabidopsis *PG* gene *AtQRT3*, indicating that these 4 *PavPGs* might function similarly with *AtQRT3* in the pectin degradation [28]. Multiple sequence alignments by Clustal X software showed that all the *PavXTH* genes contained highly conserved domain comprising of active catalytic residues and nearby potential N-linked glycosylation site (Figure 2a). Similarly, four conserved domains (motif I to IV) considered to be pivotal for PG hydrolysis activity are identified in most *PavPGs* apart from *PavPG10*, *PavPG38* and *PavPG42* (the closest ortholog of *AtQRT3*) which lack typical PG domains (Figure 2b).

### 2.3. Gene Structure, Conserved Motif and Cis-Elements Analysis of PavXTHs and PavPGs

The gene structures of *PavXTHs* and *PavPGs* were analyzed using TBtools software [35]. We found that the *PavXTHs* and *PavPGs* genes in the same phylogenetic clade show conserved exon–intron structures. The majority of *PavXTHs* had UTR structure except *PavXTH1/3/7/17/18* (Figure S2a) and almost half of the *PavPGs* had no UTR structure (Figure S2b). To further analyze the conserved motifs in the amino acid sequences of *PavXTH* and *PavPG* genes, online tool MEME was used. As shown in Figure S3a, five and eight conserved motifs were identified in *PavXTH* and *PavPG* genes, respectively. Notably, motif 1 and motif 2 are almost enriched in all *PavXTHs* except for *PavXTH7* that lacks motif 2. Similarly, motifs in PavPG genes revealed that motifs 1 and 8 are more frequently enriched (Figure S3b).

**Figure 3:**
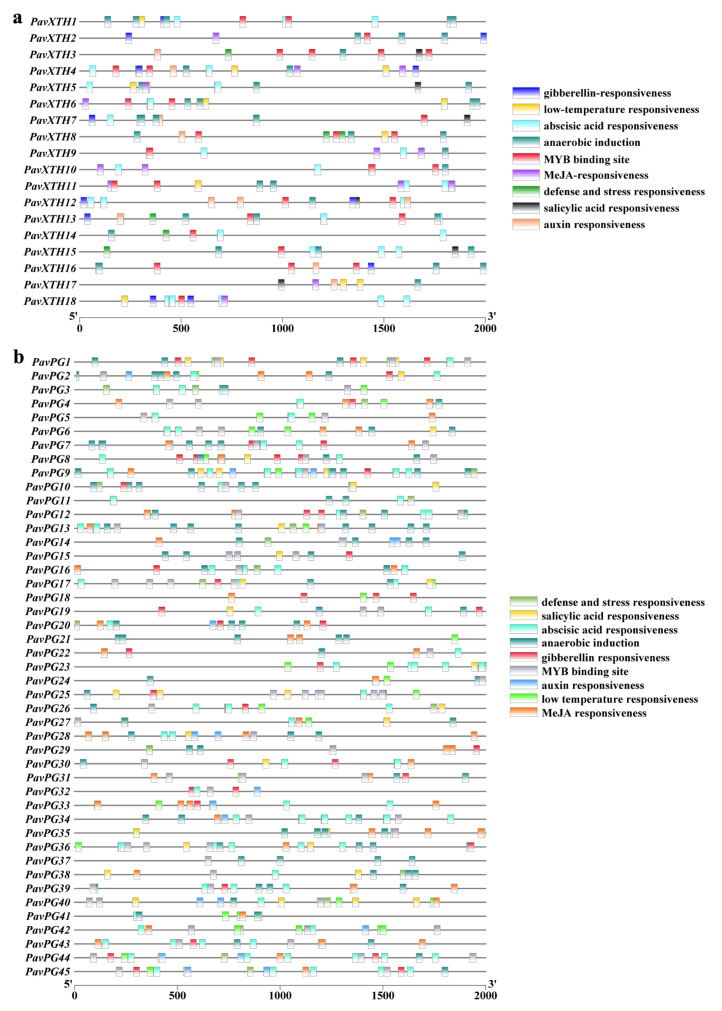
Schematic of the predicted regulatory cis-elements in the promoters of *PavXTHs* (**a**) and *PavPGs* (**b**) family genes.

Genes with similar expression profiles are likely to share common regulatory *cis*-elements in their promoters, thus we also identified cis-acting elements enrichment by utilizing the promoters of both *PavXTH* and *PavPG* genes using the PlantCARE database (Figure 3). This led to the identification of a total of 36 and 38 cis-elements located in *PavXTH* and *PavPG* promoters, respectively. Strikingly, the majority of *PavXTH* and *PavPG* genes contained the abscisic acid (ABA) responsiveness elements, emphasizing the role of ABA in regulating their expression. The remaining enriched cis-elements included defense response elements, MYB binding site, and other phytohormone-responsive elements such as auxin, MeJA, gibberellin (GA) and salicylic acid. The complexity of cis-elements distribution suggests that *PavXTH* and *PavPG* genes may participate in various biotic-abiotic/hormone signaling.

### 2.4. Identification of PavXTH and PavPG Genes Underlying Involved in Fruit Softening

**Figure 4:**
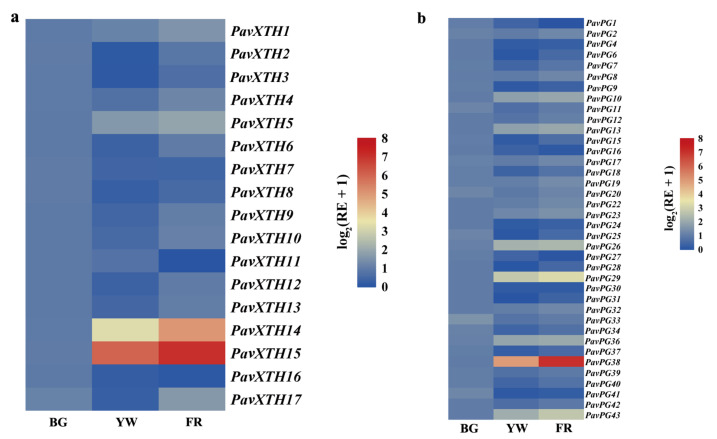
Expression patterns of *PavXTHs* (**a**) and *PavPGs* (**b**) during fruit development and ripening in sweet cherry. The colors of heatmap cells indicate the expression level of genes with log_2_ [relative expression (RE) + 1] across different samples by RT-qPCR. *PavActin* (Gene bank: FJ560908) was used as an internal control. BG, YW and FR refer to the three fruit development periods of BG (big green), YW (yellow white), and FR (full red).

**Figure 5:**
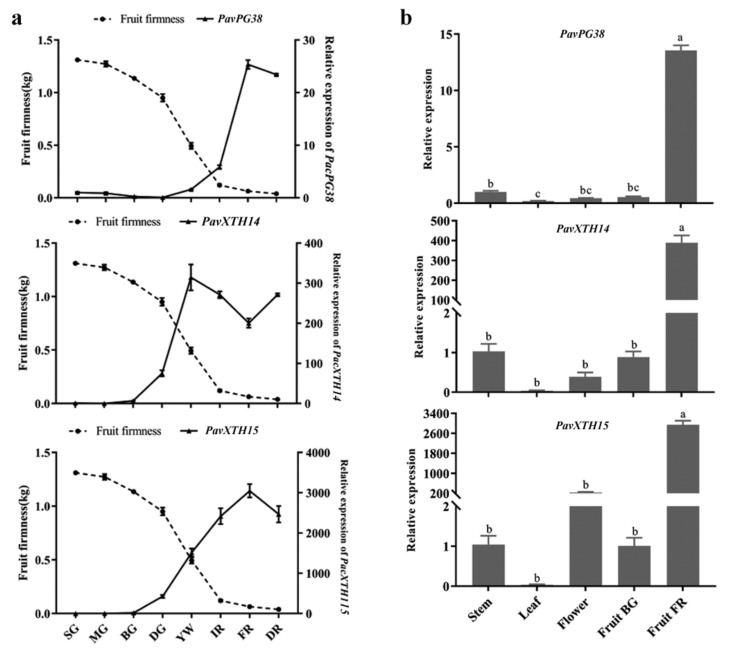
Relative expression of *PavPG38*, *PavXTH14*, and *PavXTH15* in different fruit development stages and tissues of sweet cherry. (**a**) Dynamic changes of firmness and expression of *PavPG38*, *PavXTH14*, and *PavXTH15* during fruit development, including stages of small green (SG), mid green (MG), big green (BG), degreening (DG), yellow (YW), initial red (IR), full red (FR) and dark red (DR). (**b**) Tissue-specific expression of *PavPG38*, *PavXTH14*, and *PavXTH15* in stem, leaf, flower, and fruits at stages of BG and FR. PavActin (Gene bank: FJ560908) was used as an internal control. The error bars represent the ± SD of three biological replicates. Different letters refer to significant differences by Duncan’s multiple range test with *p* < 0.05.

To further analyze the function of *PavXTH* and *PavPG* genes in fruit softening, we examined expression patterns of all these genes in sweet cherry fruit during three developmental periods of BG (big green), YW (yellow white), and FR (full red) by RT-qPCR analysis. As shown in Figure 4a,b, PavXTH and PavPG genes displayed various expression patterns during fruit development. Strikingly, expression levels of *PavXTH14*, *PavXTH15* and *PavPG38* were significantly upregulated with fruit development and ripening, indicating that they are the likely candidate genes participating in fruit softening. To investigate the potential correlation of these candidate genes and fruit softening, we compared the properties of fruit firmness and expression patterns of *PavXTH14*, *PavXTH15* and *PavPG38* at eight fruit developmental periods. As shown in Figure 5a, a converse trend of change was observed in fruit firmness and candidate gene expression. Particularly, fruit firmness decreased slowly at the early developmental periods, while rapidly decreased from degreening (DG) stage, at which *PavXTH14/15* expression starting rising substantially, indicating that DG is the key stage for softening initiation. The high level of *PavPG38* appeared in the FR period, suggesting that it mainly acts in the late stage of fruit ripening and softening. Furthermore, we also detected the spatial expression pattern of these candidate genes using different tissues. The results showed that in contrast to the vegetative tissues and flowers, three candidate genes expressed highly in the late FR fruit stage, suggesting their important role in fruit softening (Figure 5b).

### 2.5. Expression of PavXTH14, PavXTH15 and PavPG38 Gene Expression in Response to Hormones

**Figure 6:**
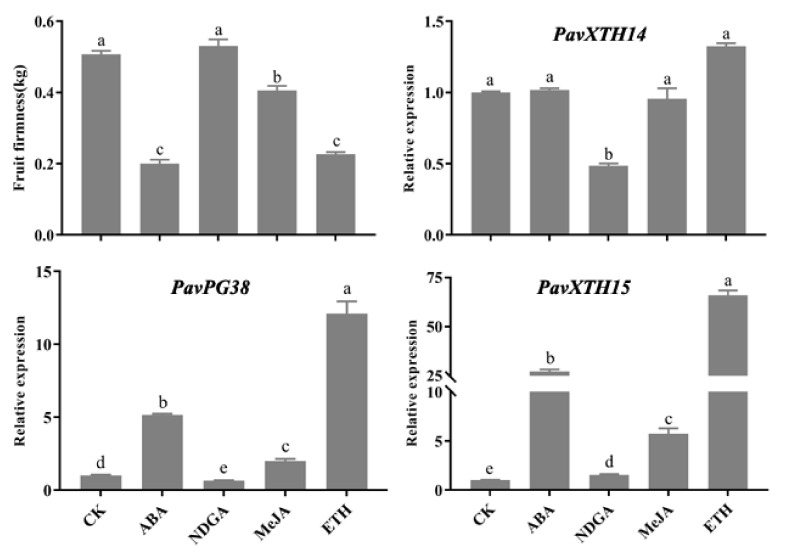
Changes in firmness and expression of *PavPG38*, *PavXTH14*, *PavXTH15* after ABA, NDGA, MeJA and ETH treatments. ABA, abscisic acid; NDGA, nordihydroguaiaretic acid; MeJA, methyl jasmonate; ETH, ethephon. *PavActin* (Gene bank: FJ560908) was used as an internal control. The error bars represent the ±SD of three biological replicates. Different letters refer to significant differences by Duncan’s multiple range test with *p* < 0.05.

As fruit ripening and softening is tightly linked to various hormone metabolism, it promoted us to test whether *PavXTH* and *PavPG* expression levels are influenced by phytohormone. After treatment with appropriate concentration of ABA, NDGA, MeJA, and ethephon in sweet cherry fruits, RT-qPCR was conducted to analyze the expression of *PavXTH14*, *PavXTH15* and *PavPG38* genes. As shown in Figure 6, ABA, MeJA and ethephon significantly promoted fruit softening. Consistently, the expression of *PavPG38* and *PavXTH15* were also upregulated by ABA, MeJA and ethephon treatments. Interestingly, ABA biosynthesis inhibitor NDGA didn’t exert any effect on fruit softening but significantly suppress the expression of *PavPG38* and *PavXTH14*. In addition, the expression of *PavXTH14* didn’t change upon ABA, MeJA and ethephon treatments. All these results suggested that the action of hormones on sweet cherry fruit softening is related to the expression levels of *PavPGs* and *PavXTHs*, at least *PavPG38* and *PavXTH15*.

### 2.6. Transient Overexpression PavXTH14, PavXTH15 and PavPG38 in Cherry Fruits

**Figure 7:**
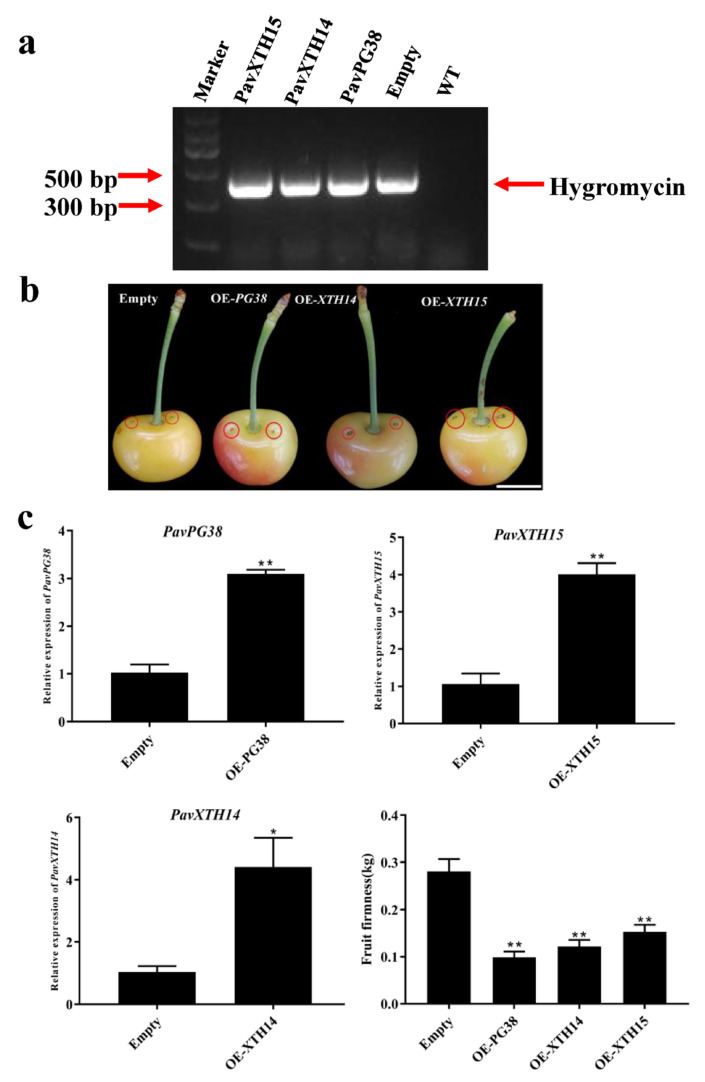
Transient overexpression of *PavPG38, PavXTH14*, and *PavXTH15* in cherry fruits. (**a**) Amplification of the 392-bp-length hygromycin DNA confirmed the working transgene system. (**b**) The phenotype of transgenic cherry fruits. The red circles indicate injection sites. (**c**) The expression of *PavPG38, PavXTH14*, *PavXTH15* and firmness in transgenic and empty control fruits. *PavActin* (Gene bank: FJ560908) was used as an internal control. The error bars represent the ± SD of three biological replicates. Significant differences in values (* *p* < 0.05, ** *p* < 0.01) were determined by Student’s *t*-test.

**Figure 8:**
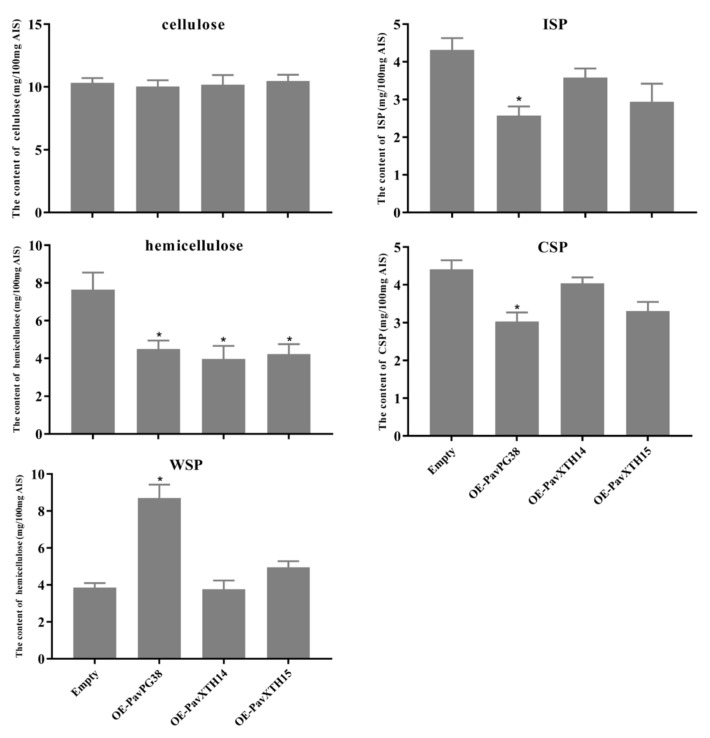
The content of cell wall compositions in transgenic fruit. CSP, WSP, ISP and AIS refer to the covalent pectin, water-soluble pectin, ion-linked pectin, and alcohol insoluble substance. The empty vector was used as a control. Values are the means ± SD from three biological replicates. Significant differences in values (* *p* < 0.05) were determined by Student’s *t*-test.

To investigate the function of candidate genes in fruit softening, we transiently overexpressed *PavXTH14, PavXTH15* and *PavPG38* in cherry fruits and analyzed its effect on firmness properties (Figure 7b). About 100 fruits were infiltrated for each gene, and after discarding all malformed fruits, 35 fruits were collected for further analysis. To confirm the availability of the transient expression system in cherry fruit, we examined the vector DNA after injecting with *Agrobacterium tumefaciens*. The positive fragment amplified from transformant suggested successful transformation (Figure 7a). Furthermore, verification of the transcript levels by RT-qPCR showed that expression of *PavXTH14, PavXTH15* and *PavPG38* in transgenic fruits were significantly higher than that of the empty control (Figure 7b). Strikingly, overexpression of *PavXTH14, PavXTH15* and *PavPG38* led to significantly reduced fruit firmness (Figure 7c). As pectin and hemicellulose are the main substrates of XTH and PG, we hypothesized that the fruit firmness change would be related to the specific cell wall structure. Therefore, three forms of pectin including covalent pectin (CSP), water-soluble pectin (WSP) and ion-linked pectin (ISP), as well as cellulose and hemicellulose in transgenic fruit were analyzed by *m*-hydroxydiphenol and anthrone method, respectively (Figure 8). As expected, the content of cellulose didn’t change, confirming that XTH and PG possess substrate specificity. In contrast, the content of hemicellulose significantly decreased in all transgenic fruit, indicating that both XTH and PG could influence the hemicellulose structure. For the analysis of pectin contents, only *PavPG38* overexpression fruit resulted in a significant alteration in pectin contents: increment of WSP, while reduction of ISP and CSP, in comparison of empty control and *PavXTH14/15* overexpression. These results suggest that *PavXTH14, PavXTH15* and *PavPG38* function as the activators of fruit softening through modification of pectin and hemicellulose.

## 3. Discussion

### 3.1. Characteristics of PavXTH and PavPG Gene Families

Given the significant role of *XTH* and *PG* genes in fruit softening identified in the past two decades, it is necessary to determine the potential functions of these genes in softening-prone sweet cherry. In this study, we systematically investigated the various bioinformatic characteristics of *PavXTH* and *PavPG* genes in sweet cherry, and a total of 18 *XTHs* and 45 *PGs* were identified. The number of *XTH* and *PG* genes varies among plant species, which may be attributed to the discrepancy in genome size and complicacy among these species. It has been showed that Arabidopsis *AtXTH* genes were classified into three clusters based on their evolutionary relationships, and this phylogenetic category has been utilized to a broad range of plant species [7,36,37]. However, the *PavXTH* genes in sweet cherry were categorized into five groups in our study. One possible explanation is the chromosomal rearrangement and genome duplications during process of evolution in sweet cherry. *TmXTH* (Gene accession: CAA48324), which was proven to be the first XTH enzyme that can hydrolyze xyloglucan molecule [38,39], was clustered together with *PavXTH14* (Figure 1a), suggesting that *PavXTH14* may have the potentiality of hydrolase to hydrolyze hemicellulose and thus resulting in fruit softening. The phylogenetic analysis showed that *PavPG* genes could be divided into seven groups, which is consistent with previous study [40]. Particularly, *PavPG10, PavPG38* and *PavPG42* in clade G were apparently different from other *PavPGs* due to the lack of any conserved PG domains. In *Arabidopsis, AtQRT3* was confirmed to degrade cell wall of the pollen mother cell during microspore development despite the absence of PG domains [28]. The high homology of *AtQRT3* (Gene accession: AY268942) with *PavPG10, PavPG38* and *PavPG42* (Figure 1b) implying that they might also possess the similar function of PG enzyme to modify the cell wall. In addition, the substantial variations of chromosomal localization, conserved domain, motif, and gene structure in *PavXTH* and *PavPG* family members suggesting their multiple functions in micro and macro environments [41].

### 3.2. Expression Profiles of PavXTH and PavPG Genes

Fruit ripening and softening are complex physiological and biochemical processes that are affected by a set of enzymes related to cell wall modification [42]. Transcriptional expression patterns and abundance of related genes during fruit development and ripening can provide vital clues for understanding their functions. It has been widely reported that the transcript levels of *PavXTHs* and *PavPGs* usually increase during fruit ripening, such as apple [43,44], strawberry [45,46], and kiwifruit [22,47]. In this study, we selected three representative periods of sweet cherry fruit to investigate the expression profiles of *PavXTHs* and *PavPGs*. The results revealed that *PavXTH14*, *PavXTH15* and *PavPG38* were significantly upregulated during fruit development and showed highest expression level in the ripening fruit (Figure 4a,b). Further detailed analysis of their expression pattern across eight fruit development stages showed that they were all upregulated along with fruit development and ripening (Figure 5a). These results provide strong evidence that *PavXTH14*, *PavXTH15* and *PavPG38* potentially participate in fruit softening of sweet cherry. In addition, the tissue specific expression analysis implied that *PavXTH15* might also function in flower development due to its high expression in the organ of flower (Figure 5b). It should be noted that the role of *PG1* in fruit softening has been confirmed in apple [26] and strawberry [27] owing to its high expression level in ripening fruits. However, in our study, *PG1* exhibited continuously low expression level across fruit development in sweet cherry (Figure 4b), implying that the function of *PG* genes is not strongly conservative among species despite they all belong to the Rosaceae plants.

It is reported that phytohormones are integral to fruit development and ripening [48]. For example, ABA has been proved to participate in fruit ripening in sweet cherry [49]. In the present study, ABA could promote fruit softening by elevating the expression of *PavPG38* and *PavXTH15* (Figure 6). In addition, exogenous MeJA treatments were quite effective in delay of fruit softening of sweet cherry [32]. Nevertheless, our results showed that MeJA increased the expression of *PavPG38* and *PavXTH15,* and thus decreased the fruit firmness (Figure 6). One explanation for this phenotypic difference is that the treatment concentration, duration, and approach are discrepant. For examples, we treated samples by soaking the fruits at YW period into the solution of MeJA for 30 min, while they selected the pre-harvest fruits before YW period to spray the MeJA on the fruit surface. Moreover, ethephon significantly reduced the fruit firmness of sweet cherry (Figure 6), implying the potential function of ethylene in regulation of fruit ripening in non-climacteric fruit.

### 3.3. PavXTH14, PavXTH15 and PavPG38 Participate in Softening by Disrupting the Fruit Cell Wall

The transient transgenic technology is increasingly applied to the verification of gene functions in fruit research [50,51]. To confirm whether candidate genes are involved in fruit softening, overexpression of these genes in sweet cherry fruit was also performed. As expected, fruits infiltrated with *PavXTH14*, *PavXTH15* and *PavPG38* exhibited firmness reduction compared to the control (Figure 7c). Recently, elevating the expression *FvXTH9* and *FvXTH6* resulted in accelerated fruit softening in strawberry fruit, and *FvXTH9* and *FvXTH6* were proved to modify the structure of xyloglucan in the cell wall [14]. In addition, overexpression of persimmon *DkXTH8* in tomato also led to cell shape changes in the transgenic fruit, intensifying fruit softening [15]. Moreover, downregulation of the *FaPG1* in strawberry caused the reduction in pectin solubilization and an increase in pectin covalently bound to the cell wall, thus resulting in delayed fruit softening [27]. All these studies suggested that *XTH* and *PG* genes are responsible for controlling fruit softening in different plant species. Consistently, our study also confirmed that *PavXTH14*, *PavXTH15* and *PavPG38* promote softening by destroying the fruit cell wall in sweet cherry. The reduced level of hemicellulose and increase of WSP in transgenic fruit indicated the destruction of hemicellulose and pectin (Figure 8), which supported the hypothesis that accelerated fruit softening is due to overexpression of these target genes.

## 4. Materials and Methods

### 4.1. Plant Materials and Treatments

The sweet cherry (*Prunus avium* L.) cultivar ‘Zaodaguo’ was grown under field conditions at Beijing Academy of Forestry and Pomology Sciences, Beijing, China. Fruit samples were collected at eight different developmental periods according to previously described [52]: small green (SG), mid green (MG), big green (BG), degreening (DG), yellow (YW), initial red (IR), full red (FR) and dark red (DR) at about 7, 10, 15, 19, 22, 25, 28, 32 days post anthesis DPA. Fruits were collected for their uniform size, same appearance, and no defects. Thirty fruits were used for firmness measurement and the left were immediately cut and frozen in liquid nitrogen and stored at −80 °C for subsequent analysis. Fruits of each stage were divided into three repeats (each repeat contained ten fruits). For tissue specific expression analysis, the stem, leaf, flower, and fruit were collected from the same tree with three biological repeats (each repeat contained ten tissues).

For phytohormone treatments, fruits at YW period were harvested from the tree and divided into five groups and soaked into water, 0.2 mM ABA, 0.2 mM NDGA, 0.5 mM MeJA, and 0.5 mM ethephon for 30 min. The water treatment was used as the control. All the solution contained 0.1% Tween-20. On the seventh day after treatments, the fruits were sampled for subsequent analysis. Fruits of each treatment (30 fruits) were divided into three repeats (each repeat contained ten fruits).

### 4.2. Mining of PavXTH and PavPG Genes in the Prunus Avium Genome

The deduced amino acid sequences of 33 *XTH* genes and 66 *PG* genes in *Arabidopsis thaliana* were used as query sequences to search against the sweet cherry genome (http://cherry.kazusa.or.jp/ accessed on 5 December 2018) by BLAST. The candidate genes were filtered and identified using the CDD database (https://www.ncbi.nlm.nih.gov/Structure/cdd/wrpsb.cgi accessed on 5 December 2018) and Pfam database (http://pfam.xfam.org/search accessed on 5 December 2018). The candidates of *PavXTH* genes that contained the two conserved domains of GH16_XET domain and XET_C domain were regarded to be *PavXTH* genes. As for *PG* genes, those genes that contained at least one highly conserved domains of the domain I, II, III, and IV of PG proteins were determined to be *PavPG* genes besides the homologous genes of *AtQRT3* (Gene accession: AY268942). The molecular weights, and isoelectric points (PI) were calculated using the ExPASy (https://web.expasy.org/protparam/ accessed on 5 December 2018). The signal peptide was predicted using the SignalP 4.1 (www.cbs.dtu.dk/services/SignalP/ accessed on 5 December 2018).

### 4.3. Phylogenetic Analysis, Multiple Sequence Alignment, Genomic Structure, Chromosomal Location, and Motif Analysis

The phylogenetic tree was constructed by the neighbor-joining (NJ) method with 1000 bootstrap replicates in MEGA 6.0 software [34]. Multiple sequence alignments were performed by Clustal X software and Espript online program (http://espript.ibcp.fr/ESPript/ESPript/index.php accessed on 5 December 2018). The gene structure was visualized by TBtools software using the GFF3 file of the sweet cherry genome. The chromosomal locations were derived from sweet cherry genome database (http://cherry.kazusa.or.jp/ accessed on 5 December 2018), and then mapped to chromosomes by TBtools software. The conserved motif was predicted through local MEME Suite (Version 5.0.5).

### 4.4. Cis-Element Prediction in the PavXTH and PavPG Gene Promoters

The promoter sequences (2 kb upstream from ATG) of *PavXTH* and *PavPG* genes were extracted from *Prunus avium* whole genome scaffolds data (version 1.0), and cis-elements in the promoters were predicted by the PlantCARE online program (http://bioinformatics.psb.ugent.be/webtools/plantcare/html/ accessed on 5 December 2018) and visualized by the TBtools software [35].

### 4.5. Measurement of Fruit Firmness and Cell Wall Materials

The fruit firmness was measured by the TA. XT Texture Analyser (Stable Microsystems, Godalming, UK). A puncture test was carried out by driving cylindrical probes (2 mm diameter) into the fruit flesh after peeling at a constant speed of 1 mm/s to a depth of 2 mm, and the maximum force during the test was recorded as the fruit firmness.

The extraction of cell wall was performed according to the previously method [1], with some modifications, 5 g of fruit flesh was ground with liquid nitrogen, add suitable dose of 95% boiling ethanol, and cook for 30 min. After cooling to room temperature, filtered the homogenate through washed successively with 50 mL 95% boiling ethanol, 50 mL chloroform: methanol (1:1 *v*/*v*), and 50 mL acetone. The insoluble substance was dried at 40 °C to get cell wall extract (alcohol insoluble substance, AIS). Weigh the AIS 100 mg, and extract the different components of cell wall in sequence as follows: 5 mL 50 mmol/L sodium acetate (pH 6.5) for water-soluble pectin (WSP); 5 mL 50 mmol/L CDTA (pH 6.5) fort ionic pectin (ISP); 5 mL 50 mM Na_2_CO_3_ (containing 20 mM NaBH_4_) for covalently bound pectin (CSP); 5 mL 4 mol/L KOH (containing 1% NaBH_4_) for hemicellulose; 1 mL 80% H_2_SO_4_ for cellulose. The pectin content was quantified the m-hydroxydiphenol method [53] with galacturonic acid as a standard. The cellulose and hemicellulose contents were quantified by the anthrone method [54] using glucose as standards.

### 4.6. RT-qPCR Analysis

Total RNA was extracted from various organs using a Plant Total RNA Extraction kit (Huayueyang Biotechnology, Beijing, China). The cDNA was synthesized using a Reverse Transcription kit (TaKaRa Biotechnology, Dalian, China). All primers are listed in Table S2. qRT-PCR was carried out using SYBR Premix Ex Taq (Kangwei Century Biotechnology, Beijing, China) with the Rotor-Gene Real-Time PCR System. All experiments were performed with three biological replicates. The relative expression of each gene was calculated using the 2^−ΔΔCt^ method (relative to *PavActin*).

### 4.7. Transient Overexpression in Cherry Fruits

The transient overexpression was carried out according to previous report [51], with slight modifications. The full-length of *PavXTH14, PavXTH15* and *PavPG38* without the stop codon were amplificated into the pCAMBIA1302 vector and transformed into *A. tumefaciens* strain EHA105. The *A. tumefaciens* suspension harboring PavXTH14, PavXTH15 and PavPG38 and the control empty vector was then separately introduced into the fruits at 20 DPA by injecting it until whole fruit was permeated. Approximately 100 fruits were infiltrated for each gene. The inoculated fruits were collected at two weeks after infiltrating. After discarding the malformed fruits, 35 fruits were sampled for following analysis. The sweet cherry cultivar ‘Rainier’ was used for this experiment. The primers are listed in Table S2.

### 4.8. Statistical Analysis

All statistical analyses were carried out using SPSS 20.0 (Windows; SPSS Inc., Chicago, IL, USA). The significance analysis was determined by the one-way ANOVA followed by Duncan’s multiple range test or Student’s *t*-test. *p* < 0.05 was considered the significant difference. *p* < 0.01 was considered the extremely significant difference.

## 5. Conclusions

In current study, we determined 18 *XTH* and 45 *PG* genes in sweet cherry and comprehensively investigate their phylogenetic relationships, conserved motifs, gene structures and the upstream regulatory cis-elements. We also analyzed the expression patterns of *PavXTH* and *PavPG* genes correlated with fruit softening. Finally, *PavXTH14*, *PavXTH15* and *PavPG38* were confirmed to participate in fruit softening by transient overexpression of these genes in sweet cherry fruits. Taken together, our genome-wide analysis of the *PavXTH* and *PavPG* gene families lays the foundation for further analyzing the roles of *XTH* and *PG* genes and provide potential targets for breeding softening-resistant sweet cherry cultivars via manipulating cell wall-associated genes.

## Figures and Tables

**Table 1:** *XTH* genes identified in Sweet Cherry.

Branch	Gene Locus	Gene Name	Deduced Protein			Signal Peptide
			Length (aa)	PI	MW (kDa)	
Ⅰ	Pav_sc0000359.1_g040.1.mk	*PavXTH1*	150	6.34	17.43	-
	Pav_sc0000308.1_g610.1.mk	*PavXTH2*	372	5.47	42.11	+
	Pav_sc0003915.1_g020.1.mk	*PavXTH3*	176	9.68	20.5	-
	Pav_sc0000428.1_g530.1.mk	*PavXTH8*	282	8.39	31.93	+
	Pav_sc0000428.1_g520.1.mk	*PavXTH9*	193	9.14	22.37	-
	Pav_sc0000428.1_g510.1.mk	*PavXTH10*	316	5.2	36.53	-
	Pav_sc0001289.1_g270.1.mk	*PavXTH13*	204	5.2	23.18	+
	Pav_sc0000212.1_g420.1.mk	*PavXTH15*	197	6.06	22.31	+
Ⅱ	Pav_sc0001450.1_g080.1.mk	*PavXTH4*	211	7.2	25.2	-
Ⅲ	Pav_sc0000480.1_g990.1.mk	*PavXTH14*	293	6.22	32.95	+
	Pav_sc0000558.1_g1030.1.mk	*PavXTH17*	639	6.44	73.43	-
Ⅳ	Pav_sc0000405.1_g520.1.mk	*PavXTH11*	287	6.89	32.51	+
	Pav_sc0000910.1_g790.1.mk	*PavXTH12*	212	4.92	24.4	+
	Pav_sc0003766.1_g160.1.mk	*PavXTH16*	277	9.41	32.62	+
	Pav_sc0006464.1_g040.1.mk	*PavXTH18*	152	7.9	17.49	-
Ⅴ	Pav_sc0000354.1_g310.1.mk	*PavXTH5*	314	7.95	35.1	+
	Pav_sc0000893.1_g780.1.mk	*PavXTH6*	168	4.98	19.01	+
	Pav_sc0001673.1_g050.1.mk	*PavXTH7*	215	5.34	24.16	-

## Data Availability

The protein sequences of XTHs and PGs gene family members from other plants were retrieved from the GenBank database (https://www.ncbi.nlm.nih.gov/genbank/).

## Supplementary Materials

The following are available online at https://www.mdpi.com/article/10.3390/ijms222212331/s1.
