# Supplementary material for: Genome-Wide Identification of the Xyloglucan endotransglucosylase/Hydrolase (XTH) and Polygalacturonase (PG) Genes and Characterization of Their Role in Fruit Softening of Sweet Cherry
Source: Int J Mol Sci. 2021 Nov 15;22(22):12331. doi: 10.3390/ijms222212331 (PMC8621145; doi:10.3390/ijms222212331)
Supplement: Supplementary file 1 [file ijms-22-12331-s001.zip › supplementary materials.pdf]

Supplementary Material

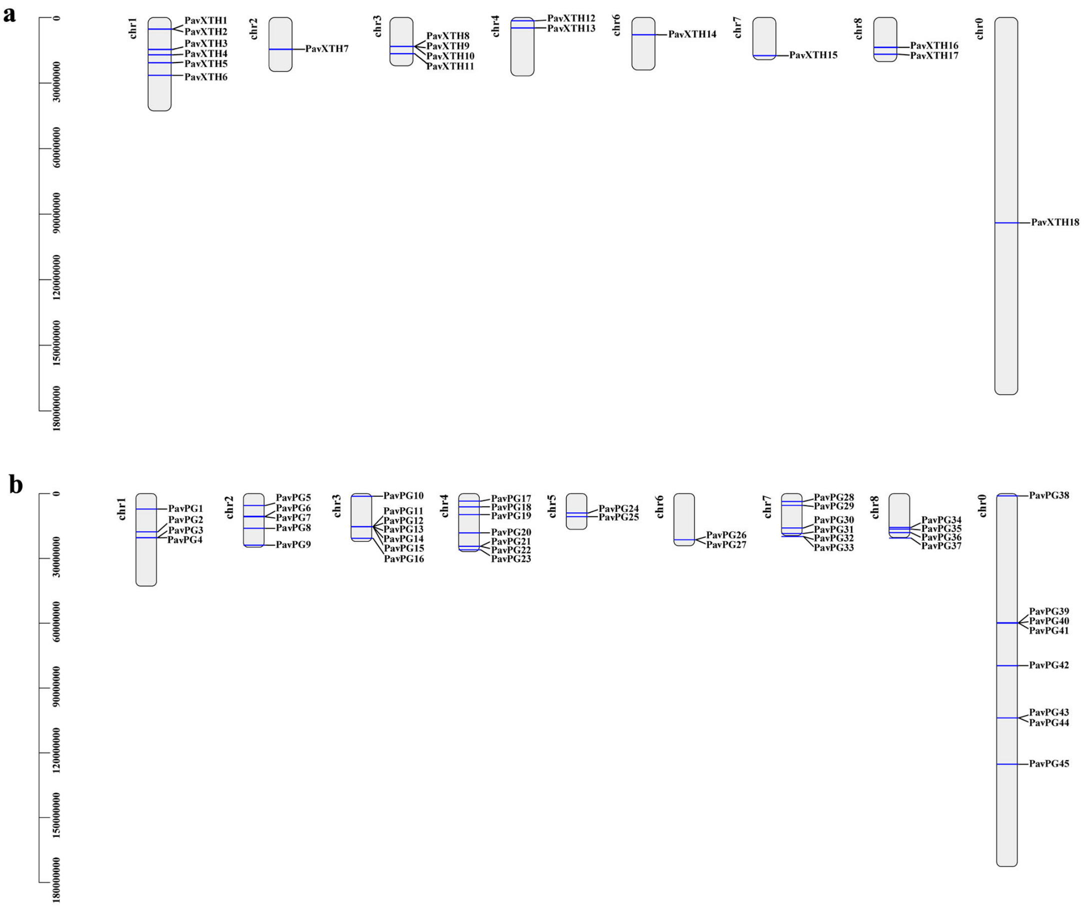

Figure S1. Chromosome distribution of sweet cherry *XTH* (**a**) and *PG* (**b**) genes. The scale on the left is in megabases (Mb).

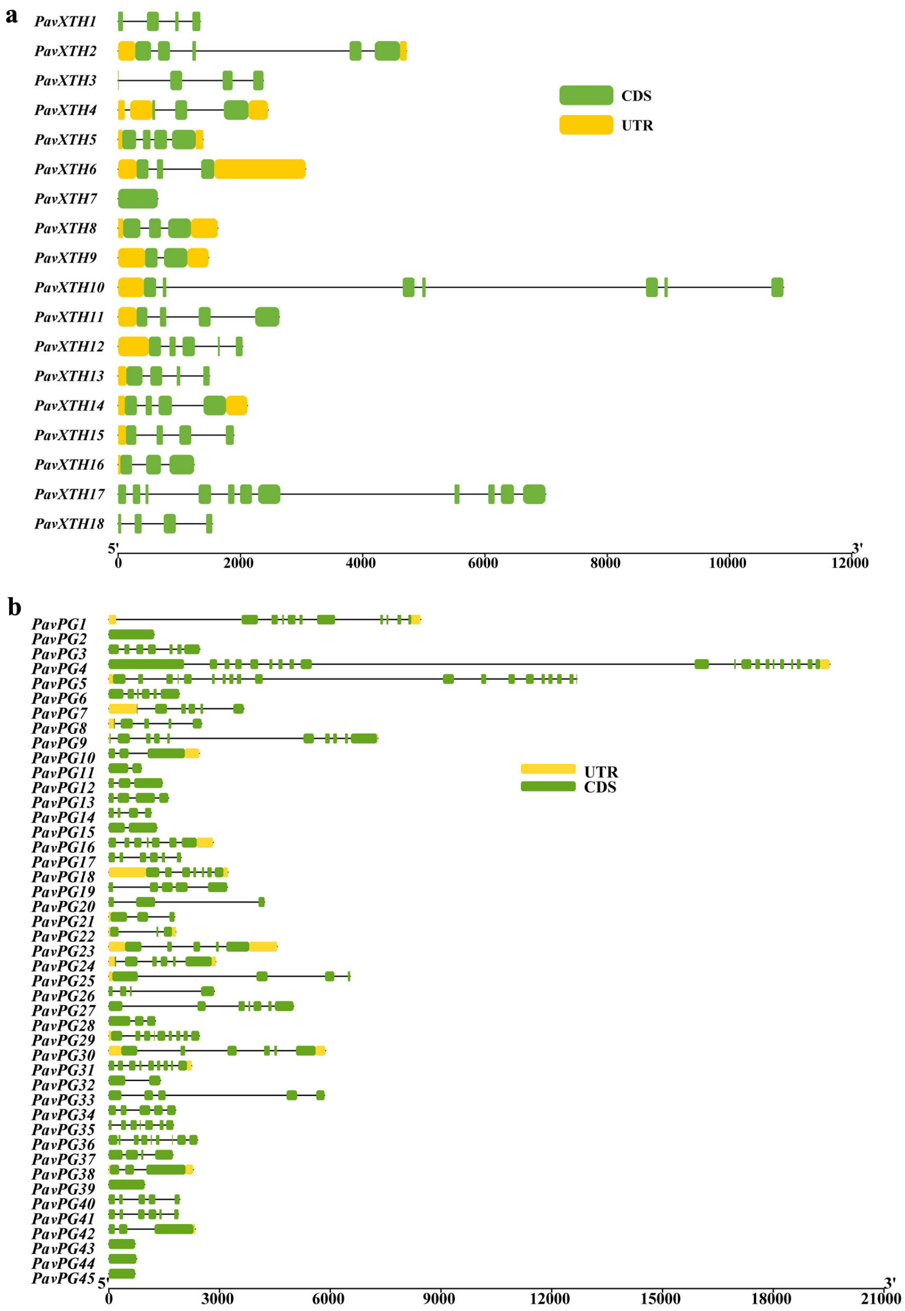

Figure S2. Gene structure analysis of sweet cherry *XTH* (**a**)and *PG* (**b**) genes.

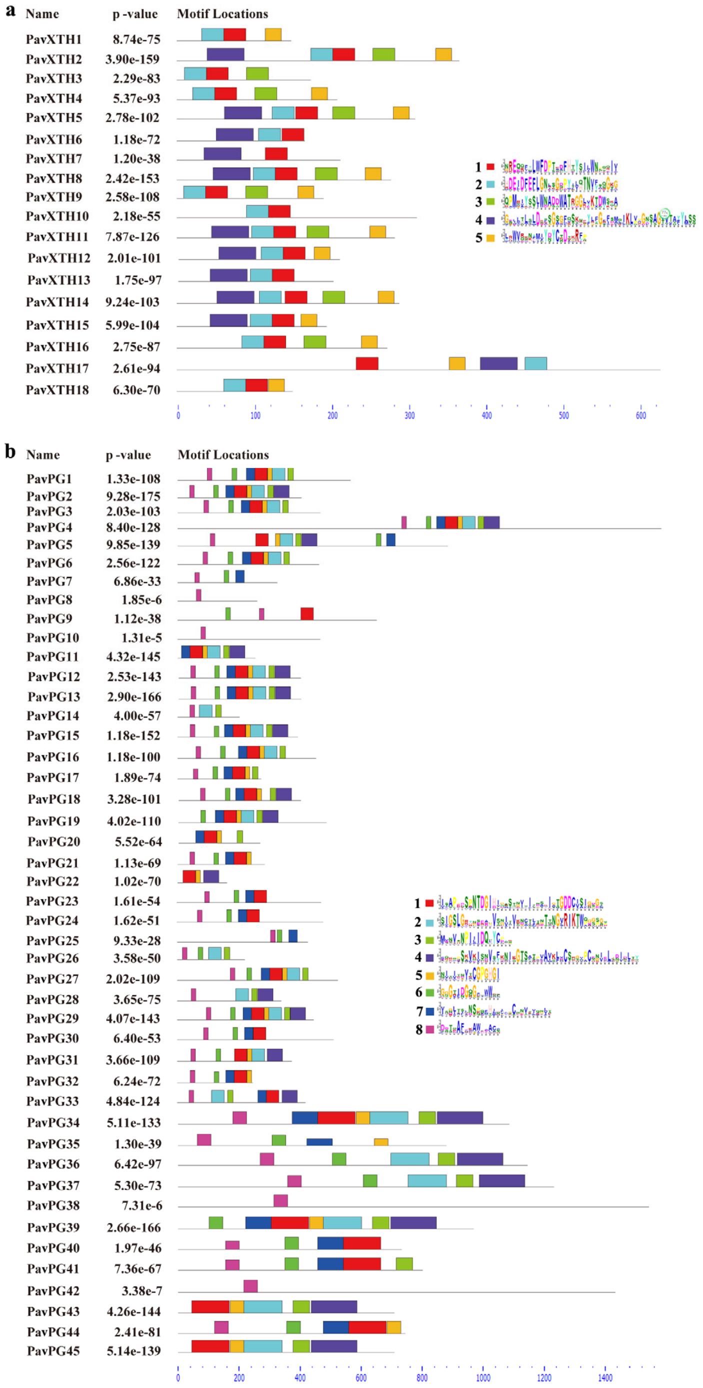

Figure S3. Conserved motif analysis of sweet cherry *XTH* (**a**) and *PG* (**b**) genes.
